# Structural Changes Due to Antagonist Binding in Ligand Binding Pocket of Androgen Receptor Elucidated Through Molecular Dynamics Simulations

**DOI:** 10.3389/fphar.2018.00492

**Published:** 2018-05-15

**Authors:** Sugunadevi Sakkiah, Rebecca Kusko, Bohu Pan, Wenjing Guo, Weigong Ge, Weida Tong, Huixiao Hong

**Affiliations:** ^1^Division of Bioinformatics and Biostatistics, National Center for Toxicological Research, U.S. Food and Drug Administration, Jefferson, AR, United States; ^2^Immuneering Corporation, Cambridge, MA, United States

**Keywords:** androgen receptor, molecular dynamics simulations, induced molecular docking, bicalutamide, agonist, antagonist

## Abstract

When a small molecule binds to the androgen receptor (AR), a conformational change can occur which impacts subsequent binding of co-regulator proteins and DNA. In order to accurately study this mechanism, the scientific community needs a crystal structure of the Wild type AR (WT-AR) ligand binding domain, bound with antagonist. To address this open need, we leveraged molecular docking and molecular dynamics (MD) simulations to construct a structure of the WT-AR ligand binding domain bound with antagonist bicalutamide. The structure of mutant AR (Mut-AR) bound with this same antagonist informed this study. After molecular docking analysis pinpointed the suitable binding orientation of a ligand in AR, the model was further optimized through 1 μs of MD simulations. Using this approach, three molecular systems were studied: (1) WT-AR bound with agonist R1881, (2) WT-AR bound with antagonist bicalutamide, and (3) Mut-AR bound with bicalutamide. Our structures were very similar to the experimentally determined structures of both WT-AR with R1881 and Mut-AR with bicalutamide, demonstrating the trustworthiness of this approach. In our model, when WT-AR is bound with bicalutamide, Val716/Lys720/Gln733, or Met734/Gln738/Glu897 move and thus disturb the positive and negative charge clumps of the AF2 site. This disruption of the AF2 site is key for understanding the impact of antagonist binding on subsequent co-regulator binding. In conclusion, the antagonist induced structural changes in WT-AR detailed in this study will enable further AR research and will facilitate AR targeting drug discovery.

## Introduction

The androgen receptor (AR), a member of the nuclear subfamily 3, is a ligand-activated transcriptional factor. AR is expressed in various tissues of different species and regulates many physiological functions including bone density, cognition, muscle hypertrophy, prostate growth and differentiation (Gelmann, 2002). AR and estrogen receptor (ER) are well characterized nuclear receptor target of active endocrine chemicals (Hong et al., 2002; Sakkiah et al., 2016). Copious experimental data and numerous *in silico* predictive models estimate both estrogenic and androgenic activity (Hong et al., 2002, 2003, 2005, 2012, 2015, 2016a,b; Shen et al., 2013; Ng et al., 2014, 2015a,b; Sakkiah et al., 2016; Ye et al., 2016). AR is a well-established drug target for prostate cancer, which is the second most common cancer by occurrence in men in western countries (Damber and Aus, 2008). Both steroid and non-steroid antagonists treat prostate cancer by blocking AR activity. A prolonged treatment course leads to tumor AR mutations, which causes AR antagonists to have a paradoxical effect. A thorough study of WT and mutant AR (Mut-AR) antagonist binding is required to better understand this paradoxical mechanism which limits therapeutic efficacy.

**FIGURE 1:**
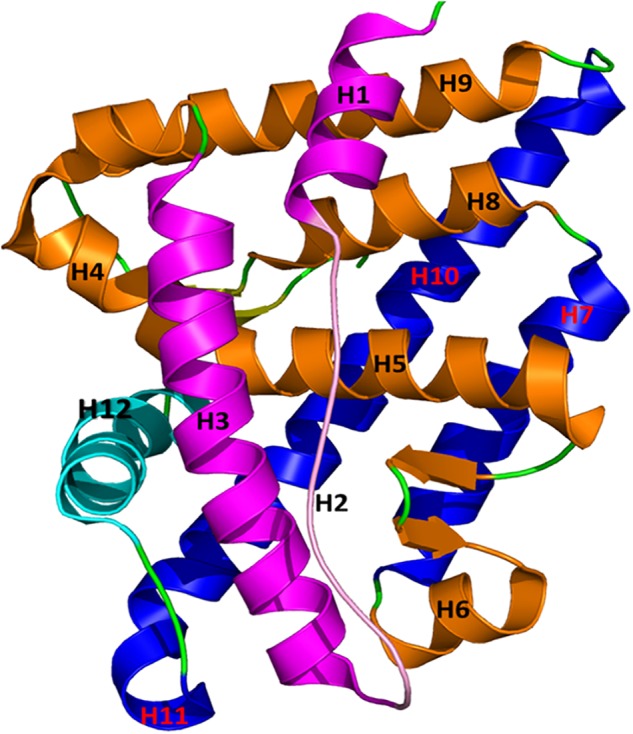
The structure of AR is plotted in a helical bundle composed of 12 helices. These helices are arranged in three layers. Layer 1 has H1, H2, and H3 (magenta), Layer 2 consists of H4, H5, H6, H8, and H9 (gold), and Layer 3 contains H7 and H10 (blue). H12 (in cyan) acts as a lid for the AR ligand binding pocket during binding of agonists and antagonists.

Full-length AR consists of 919 amino acids translated from 8 exons (Kuiper et al., 1989; Lubahn et al., 1989). Like other nuclear receptors, AR consists of three major functional domains: (1) an NH2-terminal domain, (2) a highly conserved DNA binding domain, and (3) a conserved ligand-binding domain (LBD) (Gao et al., 2005; Sakkiah et al., 2016). The hinge region acts as a bridge between the DNA binding domain and the conserved LBD. Both the AR N-terminal activation function 1 (AF1) in the DNA binding domain and the AR C-terminal activation function 2 (AF2) in the LBD control the transcriptional factors in ligand-independent and ligand-dependent manners, respectively. The AR-LBD (hereafter AR-LBD is termed as AR for simplicity) has three different binding or active sites where an agonist or antagonist can bind and alter AR functions: the ligand binding pocket, the AF2 site, and the binding function 3 (BF3) site. An agonist or a competitive antagonist can bind the AR ligand binding pocket to enhance or depress AR function, respectively. The AF2 site plays a major role in co-activator binding, which starts the transcription of AR-regulated genes. A few antagonists were reported to bind to the AF2 site, which directly blocks the binding of a co-activator protein (Axerio-Cilies et al., 2011). The BF3 site is a newly identified AR surface antagonist binding site. An antagonist can bind in any of these described binding sites to suppress AR activity. Antagonist binding causes conformational changes in the AF2 site, rendering it unsuitable for co-activators to bind AR (Estebanez-Perpina et al., 2007; Estébanez-Perpiñá and Fletterick, 2009). The three-dimensional structure of AR consists of 12 bundles of helices forming three layers (**Figure 1**). Among these 12 helices, H12 plays a major role in AR activation and undergoes a considerable conformational change due to the binding of agonist or antagonist in the ligand binding pocket. During agonist or antagonist binding, H12 functions like a “lid” which closes or moves away from the ligand binding pocket, respectively (Bohl et al., 2007; Cantin et al., 2007). When androgen binds the ligand binding pocket of AR, H12 tightly holds co-activator proteins and initiates function. AR antagonists are usually bulkier than agonists and thus require a wider binding pocket than agonists. Due to their larger size, antagonists push the residues in H12 (which is near the ligand binding pocket) outward to expand the active site. These structural changes in the ligand binding pocket cause the AF2 site to undergo conformational changes, preventing co-activator protein binding (Estébanez-Perpiñá and Fletterick, 2009). Some mutations in AR cleverly cause drug resistance by converting AR antagonist properties into agonist properties. Prostate cancer drug resistance is predominantly driven by AR mutations. For example, mutations T877A (Sack et al., 2001; Bohl et al., 2007), W741L/C (Hara et al., 2003), L701A/T877A (Balbas et al., 2013), and F878L (Balbas et al., 2013; Korpal et al., 2013) in the LBD made AR antagonists Flutamide, R-bicalutamide, and Enzalutamide behave as agonists. The mutation T877A significantly increased the activity of AR, as evidenced by the enhanced AR affinity toward progesterone and estrogens (Taplin and Balk, 2004).

There exist 90 crystal structures of AR from different species (rat, mice, chimpanzee, and human) in the Protein Data Bank (PDB^1^) (Berman et al., 2000). Wild type AR (WT-AR) crystal structures exist with either agonists in the ligand binding pocket or antagonists in the AF2 or BF3 sites. Mut-AR crystal structures exist with antagonists in the ligand binding pocket. No 3D structure of WT-AR with an antagonist in the ligand binding pocket has been described, likely because an antagonist binding to the AR-chaperone complex does not disassociate the chaperone from AR (Bohl et al., 2005; Sakkiah et al., 2016). To fill this knowledge gap, the AF2 site structural changes in WT-AR which are induced by antagonist binding could be determined via molecular modeling.

Determining the conformation change of a protein induced by a ligand using crystallography is at best time consuming but often infeasible. Several researchers employed molecular dynamics (MD) simulations to characterize H12 structural changes due to antagonists or agonist binding in the AR ligand binding pocket. Zhou J. et al. (2010) utilized replica-exchange MD to characterize structural conformational changes and H12 movement caused by binding of hydroxyflutamide in the ligand binding pocket of WT and mutant (T877A) AR. Using MD simulations, Bisson et al. (2008) proposed that T877A in AR destabilized hydroxyflutamide–Met895 interactions and thus decreased hydroxyflutamide antagonist activity. Additionally, Osguthorpe and Hagler (2011) employed MD simulations and quantum mechanics to discover that an antagonist occupied more space than an agonist, leading to H12 instability. While important contributions to the field, these MD simulations were limited by short time frames and mainly focused on the ligand binding pocket or H12 structural changes (Bisson et al., 2008; Osguthorpe and Hagler, 2011; Liu et al., 2015, 2016, 2017; Wang et al., 2017). Recently, many researchers captured structural changes of various proteins using long time MD simulations (hereafter called “long MD simulations”) (Whitten et al., 2005; Dror et al., 2009; Khelashvili et al., 2009; Nury et al., 2010; Gotz et al., 2012; Durrant et al., 2016). For example, Lindorff-Larsen et al. (2011) predicted the folding of 12 proteins using MD simulations ranging from microsecond to a millisecond. Their results unveiled a common principle for the folding of the 12 structurally diverse proteins and more importantly demonstrated that long MD simulations are a power tool to predict and capture protein conformational changes (Lindorff-Larsen et al., 2011). Next, Kumar and Purohit (2014) found that the long MD simulations significantly increased prediction accuracy when studying cancer associated single nucleotide polymorphisms. Thus, long MD simulations overcome many limitations of short-term MD simulations. Duan et al. (2016) conducted 1 μs MD simulations and explored ligand binding pocket changes during agonist and antagonist binding in WT and Mut-AR. Using bias-exchange meta-dynamics to study the free energy profile of agonist and antagonist binding to AR, they observed agonist and antagonist binding driven movement of H12 and structural changes in the ligand binding pocket of WT-AR. They also reported that long MD simulations were required to capture H12 movement, whereas short-term stimulations miscalculated agonist binding induced H12 structural changes (Duan et al., 2016). Hence, in this study, we applied long MD simulations (1 μs) not only to capture H12 movement but also to study AF2 site structural changes due to antagonist binding in the AR ligand binding pocket.

Three AR complex structures were studied to understand the antagonist binding induced structural changes of the AF2 site. R1881 and bicalutamide are, respectively, well-known as an agonist and an antagonist for AR. Structures of AR bound with R1881 and bicalutamide were downloaded from PDB: WT-AR-R1881 (AR with agonist, PDBID: 1E3G) and Mut-AR-bicalutamide (AR with antagonist, PDBID:1Z95). The third AR complex structure, WT-AR-bicalutamide, was absent from PDB and thus was generated using the induced fit molecular docking (IFD) method (explained in the Section “Materials and Methods”). The IFD method explores both possible binding poses of a ligand in a receptor active site as well as the associated conformational changes of the side chains near the active site. MD simulations are an important tool to study receptor–ligand interactions at an atomic level for a given time frame. MD simulations optimize three-dimensional complex protein structure bound with a ligand obtained from X-ray crystallography or molecular docking. Here, we leveraged the advantages of IFD and MD simulations together to understand the subtle structural changes in WT-AR due to anti-androgen binding and also to elucidate key co-activator binding residues in the WT-AR AF2 site. Each AR complex structure was subjected to 1 μs of MD simulations to resolve important AF2 site residue reformation during the binding of small molecules in WT-AR. Our results will enable design of improved prostate cancer treatments and facilitate endocrine disruption chemical risk assessment through AR-mediated responses.

## Materials and Methods

### Molecular Docking

Rigid docking (only giving flexibility to ligands) might fail to produce a precise ligand pose due to rigidness of the protein. In contrast, IFD gives flexibility to adjust not only the active site but also the side chain orientations of the protein to fit the pose and conformation of the bound ligand (Zhong et al., 2009). Hence, it can generate many protein-ligand complexes by changing the side chains or the backbone of the protein. Glide (docking) and Prime (refinement) modules were used in the IFD to determine the possible binding modes of the ligand and the concomitant binding induced conformational changes.

The IFD (Sherman et al., 2006a,b) module^2^ from the Schrodinger-Suite (2016b) was used to dock the AR antagonist, bicalutamide, in WT-AR.

The following steps were involved in the IFD employed here (Wang et al., 2008; Luo et al., 2013):

(i) The protein was refined using the Protein Preparation module.
(ii) Each ligand was docked (Glide module) in a defined region using a softened potential to produce 20 different poses (default setting).
(iii) A sidechain prediction (Prime module) within a given distance of the ligand was conducted for each complex.
(iv) The defined region of the protein-ligand complexes was minimized.
(v) The refined protein-ligand complexes were re-docked using Glide by specifying the lowest energy structure.
(vi) The IFD score (binding energy) was calculated for each complex.

Protein preparation is one of the most important steps in molecular docking and plays a key role in IFD. The three-dimensional atomic coordinates of WT-AR (PDB ID: 1E3G) (Matias et al., 2000) were retrieved from PDB and used as a receptor for the IFD. The Protein Preparation module^3^ was used to add hydrogen atoms and to build the missing side chains, residues, and loops. The OPLS-2001 force field (Jorgensen and Tirado-Rives, 1988; Kaminski et al., 2001; Shivakumar et al., 2010) was used to assign the partial charges. All water molecules were removed and the protein structure was optimized using the OPLS force field. A 10 Å docking grid was generated around the ligand, R1881, in WT-AR. The structure of bicalutamide was obtained from the crystal structure of Mut-AR-bicalutamide (PDB ID: 1Z95) (Bohl et al., 2005) and docked in the generated grid box using Glide XP docking. The Glide XP docking (Halgren et al., 2004; Friesner et al., 2006; Shelley et al., 2007) generated 20 different bicalutamide poses for the WT-AR structural refinements. The Prime module was used to refine the generated WT-AR-bicalutamide complexes. In the Prime refinement, each WT-AR-bicalutamide conformation from the previous step was subjected to side chain and backbone refinements (Jacobson et al., 2004) by selecting the residues within 10 Å from bicalutamide and/or residues from 669 to 918. The Prime energy was calculated and used to rank the refined AR-bicalutamide complexes. The lowest energy conformation (30 kcal/mol) of the refined WT-AR complex was used to re-dock the bicalutamide using Glide XP mode. The most favorable binding pose of bicalutamide in WT-AR was selected based on the IFD score (binding energy). The selected WT-AR-bicalutamide complexes were visualized to check the interactions between bicalutamide and the residues in the ligand binding pocket using Ligand Interactions module in Maestro 11 (Schrodinger-Suite, 2016a).

### Molecular Dynamics Simulations

Proteins are dynamic in nature. Thus, understanding atomic level motion is required to capture their profound dynamic mechanisms (Chou and Mao, 1988; Chou et al., 1994; Wang and Chou, 2009). MD simulations have the capacity to analyze the dynamics of an apoprotein or a complex with other molecules in an aqueous environment (Sakkiah et al., 2013a,b). Moreover, MD simulations yield energetically favorable conformations by optimizing a protein-ligand complex, which is needed to understand protein–ligand interactions and ligand binding induced structural changes.

The structures of the WT-AR-bicalutamide complex (obtained from IFD), WT-AR-R1881, (PDBID: 1E3G) (Matias et al., 2000), and the Mut-AR-bicalutamide complex (PDBID: 1Z95) (Bohl et al., 2005) were subjected to MD simulations using the Amber 14 package (Case et al., 2005). Then the topology and coordinate files for the agonist and antagonist were prepared using antechamber. Tleap was used to prepare the topology and coordinate files for the protein as well as to make the AR complex for running MD simulations. Amber03 molecular mechanical force field (Duan et al., 2003) and general AMBER force field (gaff) (Wang et al., 2004) were employed for the protein and ligands (agonist and/or antagonist), respectively. Each of the complex structures were immersed into a rectangular box of TIP3P model water (Jorgensen et al., 1983). The boundaries of the water box size were 10 Å away from the nearest atoms of the complex. All systems were neutralized by adding Cl^-^ ions. The Particle Mesh Ewald (PME) (Darden et al., 1993) and SHAKE (Ryckaert et al., 1977) algorithms were used to handle long-range electrostatic interactions for all heavy and hydrogen atoms involved in the covalent bonding. A cutoff of 10 Å was used for the short-range interactions (van der Waals and electrostatic interactions). In the first phase, only the solvents were minimized and equilibrated inside the water box. Then, the whole system was minimized and equilibrated by applying the steepest descent minimization for 1000 cycles, followed by conjugate gradient energy minimization for 4000 cycles. Subsequently the whole system was gradually heated from 0 to 310.15 K over a 100 ps period which was followed by a 250 ps equilibrium simulation for the whole systems. In the second phase, the prepared systems were subjected to 1 μs of MD simulations using Amber14. All MD simulations were performed with a time step of 2 fs. The coordinates were saved for every 1 ps. MD simulations were performed using PyMol (Schrodinger, 2015) and Visual Molecular Dynamics (Humphrey et al., 1996). The Amber package^4^ was used to calculate RMSD values for the protein and ligands as well as RMSF values for residues.

## Results and Discussion

### IFD Produced a Structure of WT-AR-Bicalutamide for MD Simulations

No crystal structure for WT-AR with an antagonist in the ligand binding pocket has been deposited in PDB (accessed on May 19, 2017). To address this open question, we conducted IFD. Flexibility was given to the active site residues and the ligand during Glide docking. The whole WT-AR-bicalutamide system was refined using the Prime module to predict the suitable binding orientation of bicalutamide in the ligand binding pocket of WT-AR. Among the 20 models generated for WT-AR-bicalutamide, the top 5 models were selected based on their IFD/Glide scores and checked for residue interactions (**Table 1**). Among these 5 complex structures, Model-1, Model-3, and Model-4 showed a π–cation interaction with Trp741 and Phe764. Trp741 had van der Waals interactions favorable for agonist binding in the ligand binding pocket of WT-AR (Bohl et al., 2005). In contrast, Model-2 and Model-5 failed to form π–cation interactions with Trp741 or Phe874. Model-3, Model-4, and Model-1 had shown three, three, and two hydrogen bond interactions between bicalutamide and WT-AR, respectively. In Model-3 and Model-4, bicalutamide formed hydrogen bond interactions with Leu704, Asn705, and Arg752. Importantly, the hydrogen bond between the agonist/antagonist with Arg752 in WT-AR is crucial for AR activity (Gao et al., 2005; Bohl et al., 2007; Tan et al., 2015). Bicalutamide in Model-1 failed to form hydrogen bond interactions with Arg752. Model-3 had a better binding affinity value than Model-4. Interestingly, bicalutamide in Model-3 showed a bent conformation, which is different from the bicalutamide conformation in the Mut-AR (Gao et al., 2005). Previous evidence proposed that bicalutamide forms a hydrogen bond with residues Arg752, Leu705, Asn705, and Gln711 in Mut-AR (Tan et al., 2015). While Model-3 also formed a hydrogen bond with critical residues (Leu704, Asn705, and Arg752) it failed to form a hydrogen bond with Gln711 and did not adopt a similar pose with the agonist due to the bulkier tryptophan side chain. Additionally, in Model-3, the 4-fluorophenyl group of bicalutamide moved toward the H12 region to form a suitable position in the WT-AR ligand binding pocket. Hence, Model-3 was selected for subsequent MD simulations of WT-AR-bicalutamide based on IFD score and binding interactions.

**Table 1:** Induced fit docking (IFD) score and the key residues involved in hydrogen bond interactions between WT-AR and bicalutamide for the top 5 complexes.

Model #	Glide score	IFD score	Interactions	
			Hydrogen bond	π–Cation
Model-1	-12.75	-600	Leu704, Asn705	Trp741, Phe764
Model-2	-12.11	-600	Leu704, Asn705	Trp741
Model-3	-13.01	-600	Leu704, Asn705, Arg752	Trp741, Phe764
Model-4	-11.78	-598	Leu704, Asn705, Arg752	Trp741, Phe764
Model-5	-11.20	-598	Leu704, Asn705	Phe764

### System Stability and Fluctuation Analysis Revealed Stability of AR Structures

**FIGURE 2:**
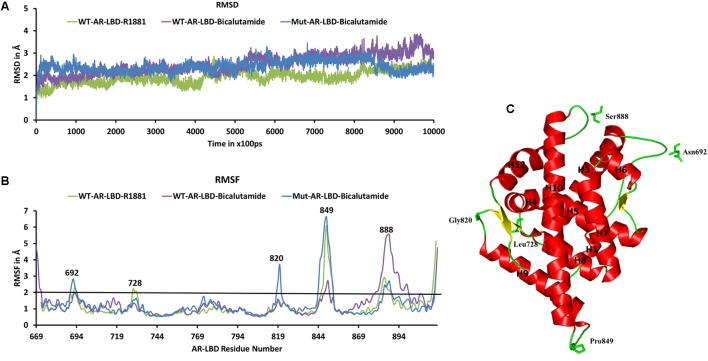
**(A)** Shows the root mean square deviation (RMSD) plot of the systems during the 1 μs MD simulations. The RMSD values were calculated using AR backbone atoms. The *X*-axis represents time with a unit of 100 ps and the *Y*-axis shows RMSD values in Å. **(B)** Shows the root mean square fluctuation (RMSF) of the Cα atoms of AR systems in the 1 μs MD simulations. The *X*-axis indicates AR residue number and *Y*-axis represents RMSF in Å. The residues with RMSF > 2 Å are marked. **(C)** Demonstrates the structure of WT-AR-R1881, residues with RMSF > 2 Å in the loop regions are marked. These residues are drawn in a stick model. WT-AR-R1881 is color coded in green, WT-AR-bicalutamide in purple, and Mut-AR-bicalutamide in blue.

We used the three molecular systems listed in **Table 2** (WT-AR-R1881, WT-AR-bicalutamide, and Mut-AR-bicalutamide) to analyze the structural changes in WT-AR due to bicalutamide binding in the ligand binding pocket using MD simulations. All trajectory files obtained from the MD simulations were examined for stability and fluctuation of the systems. Metrics of root mean square deviation (RMSD) and root mean square fluctuation (RMSF) were calculated for all systems to measure their energetic stability and the spatial fluctuation of residues, respectively. **Figure 2A** plots the RMSD values of the three systems during the 1 μs simulations. The RMSD values converged in the last 100 ns, indicating that the systems had reached a stable state. The WT-AR-R1881 and Mut-AR-bicalutamide systems were stabilized with an RMSD value of around 2.0 Å, while the WT-AR-bicalutamide system had a higher RMSD value of about 2.5 Å. An average structure was calculated from the last 100 ns for each of the three systems. The structure with the lowest RMSD value compared with the average structure in last 100 ns was selected as a representative structure for each of the systems to elucidate the structural changes of WT-AR induced by bicalutamide.

**Table 2:** Three molecular systems in MD simulations.

#	PDB ID	Ligand	System
1	1E3G	R1881	WT-AR-R1881
2	1Z95	Bicalutamide	WT-AR-bicalutamide
3	1Z95	Bicalutamide	Mut-AR-bicalutamide

Root mean square fluctuation plots were used to analyze flexibility of the residues in AR in the 1 μs MD simulations. Examination of the RMSF plots in **Figure 2B** revealed that WT-AR-bicalutamide had a larger RMSF value compared with WT-AR-R1881 and Mut-AR-bicalutamide near the C-terminal of LBD (mostly near H12). The average RMSF value for WT-AR-bicalutamide, Mut-AR-bicalutamide, and WT-AR-R1881 was 1.29, 1.25, and 1.11 Å, respectively. Five residues (Asn692, Leu728, Gly820, Pro849, and Ser888) in AR had an RMSF of >2.0 Å (**Figure 2B**) and were considered to be flexible residues. These five residues were present in the loop region of AR (**Figure 2C**). The RMSF values of the active site residues were small, demonstrating the stability of the AR active site.

### Key Structural Changes in WT-AR Binding Antagonists

**FIGURE 3:**
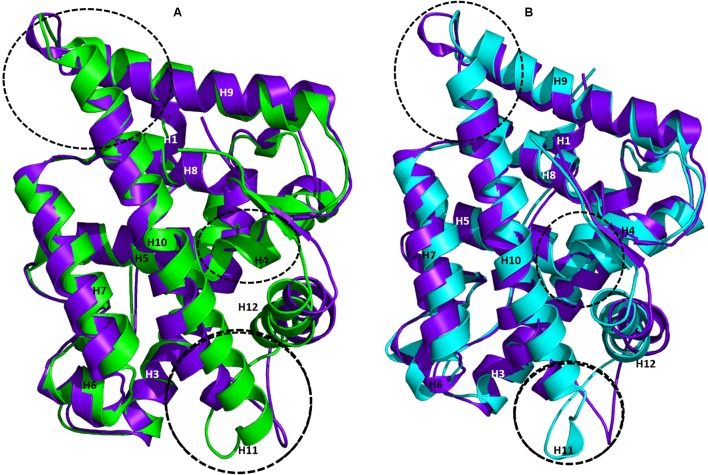
Superimposition of WT-AR-R1881 and WT-AR-bicalutamide **(A)** and superimposition of WT-AR-bicalutamide and Mut-AR-bicalutamide **(B)**. AR-R1881 is drawn in green, WT-AR-bicalutamide in purple, and Mut-AR-bicalutamide in cyan. The black dotted circles mark the structural changes between the two structures.

The AR ligand binding pocket accommodates both agonists and antagonists. Most antagonists bind in this site and alter the function of AR. The representative structures of WT-AR-R1881 and WT-AR-bicalutamide obtained from the MD simulations were superimposed to examine the difference between the two systems. Several major structural changes were identified in WT-AR due to the bicalutamide binding compared with agonist binding (R1881) (**Figure 3A**). Comparison of WT-AR-bicalutamide with WT-AR-R1881 showed a distortion at the end of H10 due to bicalutamide binding. Several residues in H10 were changed into a loop, which enabled more flexible movement. The structural conversion of H11 into a loop moved H12 away from the AR ligand binding pocket. Moreover, structural changes were observed when comparing WT-AR and Mut-AR bound with bicalutamide (**Figure 3B**). During bicalutamide binding, H11 was retained in the Mut-AR structure but was changed into a loop in the WT-AR structure (marked by the dotted circle in **Figure 3B**). As expected, Mut-AR-bicalutamide had a similar 3D structure to WT-AR-R1881.

The ligand binding pocket area and volume were calculated using the online Computed Atlas of surface Topography of protein server^5^. The area/volume for WT-AR-R1881, WT-AR-bicalutamide, and Mut-AR-bicalutamide were 185/90, 528/321, and 366/193, respectively. As expected, area and volume of the ligand binding pocket of WT-AR-bicalutamide were larger than the agonist binding in WT-AR and bicalutamide binding in Mut-AR. Bicalutamide is larger than R1881 and hence moved H12 outward from the ligand binding pocket. The RMSD values comparing the WT-AR-R1881 vs. WT-AR-bicalutamide as well as WT-AR-bicalutamide vs. Mut-AR-bicalutamide were calculated for each residue by superimposing the structures using Visual Molecular Dynamics (Humphrey et al., 1996). The residues were ranked based on the computed RMSD values and are plotted in Supplementary Figure S1. The RMSD values showed a gap between 2.8 and 3 Å in both comparisons (Supplementary Figures S1A,B). There were 42 and 37 residues with RMSD value greater than 2.8 Å between WT-AR-R1881 and WT-AR-bicalutamide and between WT-AR-bicalutamide and Mut-AR-bicalutamide, respectively. These residues are summarized in Supplementary Tables S1, S2. Twenty-two WT-AR-R1881 vs. WT-AR-bicalutamide residues and 26 WT-AR-bicalutamide vs. Mut-AR-bicalutamide residues were in helices (H3, H7, H9, H10, and H12), while the other residues were in loop regions.

The Trp741 mutation played a major role in the conversion of an AR antagonist into an agonist. The flipped Trp741 side chain moved His874 in H10 away from the ligand binding pocket to accommodate bicalutamide. Leu873, Phe876, Thr877, and Met895 were the active site residues in the ligand binding pocket showing RMSD values greater than 3 Å between WT-AR-R1881 and WT-AR-bicalutamide. Thr850, Ser851, His874, Phe878, and Leu881 from H10 also had RMSD values greater than 3 Å (Supplementary Table S1). These structural changes drove the ligand binding pocket of WT-AR to expand to accommodate bicalutamide.

The representative structure of WT-AR-R1881 superimposed well with Mut-AR-bicalutamide compared with the superimposition of WT-AR-R1881 and WT-AR-bicalutamide. The H12 residues in Mut-AR-bicalutamide were not very different from the H12 residues in WT-AR-R1881. All residues in Mut-AR had less than 2.5 Å RMSD compared with WT-AR-R1881. Mut-AR-bicalutamide additionally did not experience large structural changes compared to WT-AR-R1881. The mutant residue Trp741Leu in Mut-AR-bicalutamide had a similar conformation to the wild type residue in WT-AR-R1881. The residues showing RMSD greater than 2.8 Å between WT-AR-bicalutamide and WT-AR-R1881 are listed in Supplementary Table S1.

Lastly, Mut-AR-bicalutamide and WT-AR-bicalutamide representative structures were superimposed to identify the crucial residues that played important roles in bicalutamide binding to AR. H11 in WT-AR-bicalutamide changed into a loop. The residues 882–984 in the loop region between H10 and H12 gave more flexibility for H12 to move away from the ligand binding pocket in WT-AR-bicalutamide. All these residues had RMSD values greater than 3.5 Å compared with WT-AR-R1881. Notably, the residues from His885 to Asp890 had RMSD values greater than 6 Å. These residues forming H11 in Mut-AR-bicalutamide reduced the flexibility of the loop and held H12 close to the ligand binding pocket. As expected, these residues showed RMSD values less than 2.8 Å between WT-AR-R1881 and Mut-AR-bicalutamide. Hence, we posit that the structural change of H11 into a loop in WT-AR-bicalutamide plays an essential role in H12 movement and thus makes the AF2 site not suitable for co-activator binding. The residues which are different between Mut-AR-bicalutamide and WT-AR-bicalutamide are listed in Supplementary Table S2.

**FIGURE 4:**
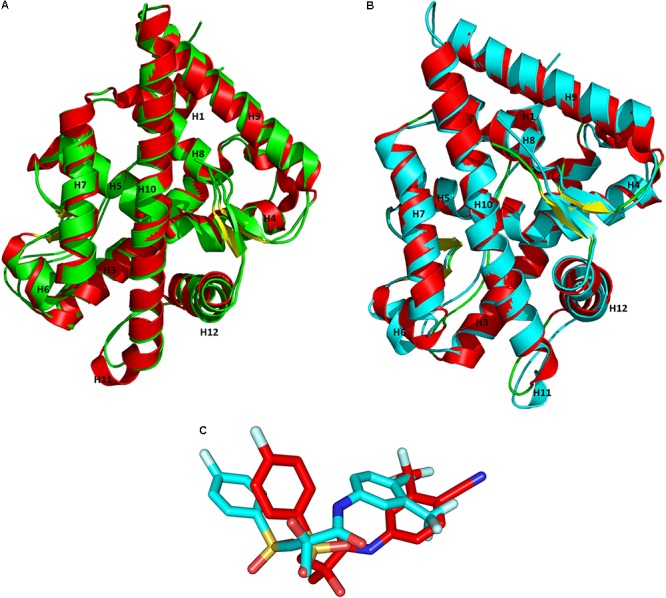
Superimposition of the representative structures from the MD simulations and the X-ray crystal structures from PDB for WT-AR-R1881 **(A)** vs. Mut-AR-bicalutamide **(B)**. The protein is drawn as a ribbon model. Overlay of bicalutamide structures from PDB are in red and the calculated WT-AR are in cyan **(C)**. The X-ray crystal structure of AR is colored in red, the representative structure of WT-AR-R1881 in green, and Mut-AR-bicalutamide in cyan.

Superimposition of the X-ray crystal structures and the representative structures from our MD simulations had an RMSD value of 1.10 Å for WT-AR-R1881 (**Figure 4A**) and 1.02 Å for Mut-AR-bicalutamide (**Figure 4B**). This indicates that the selected representative structures do not deviate much from the X-ray crystal structures. Furthermore, the orientations of R1881 and bicalutamide were also similar to the crystal structures. The overlay of bicalutamide from the Mut-AR X-ray crystal structure and the representative WT-AR structure from MD simulations had an RMSD value of 5.2 Å (**Figure 4C**). This comparative analysis confirmed that the representative structures of WT-AR-bicalutamide obtained from the MD simulations are reliable and were not obtained by chance. Therefore, the representative structure of WT-AR-bicalutamide could be reliably used to elucidate the structural changes in WT-AR due to antagonist binding.

### Identification of Critical Residues in the AF2 Site

The AR AF2 site is bound by co-activator proteins, which initiates the transcription of target genes. **Table 3** lists the important residues in WT-AR and their interactions with co-activator proteins (Askew et al., 2007; Estebanez-Perpina et al., 2007; Hsu et al., 2014). The interactions between AR and co-activators were identified from 17 WT-AR-agonist and two Mut-AR-agonist complexes in the PDB. Most of the residues (Val713, Val716, Lys717, Lys720, Phe725, Val730, Gln733, Met734, Ile737, Gln738, Glu893, Met894, and Ile898) in the AF2 site formed hydrophobic interactions with co-activator proteins. Five residues (Val716, Met734, Ile737, Gln738, and Met894) in the AF2 site had hydrophobic interactions with most of the co-activators. Glu897, Lys720, Asp731, and Gln733 formed hydrogen bond interactions with co-activator proteins and Glu897 and Lys720 formed hydrogen bond interactions with most of the co-activators (Askew et al., 2007; Estebanez-Perpina et al., 2007; Hsu et al., 2014). From the structural analysis, it was clear that Val716, Met734, Ile737, Gln738, Met894, Glu897, and Lys720 played a paramount role in tight binding of co-activator proteins.

**Table 3:** Critical WT-AR AF2 site residues involved in the hydrophobic and hydrogen bond interactions with a co-activator.

PDB ID	Mutation	Hydrophobic interaction	Hydrogen bond interaction
2PKL (Estebanez-Perpina et al., 2007)		Val716, Lys720, Gln733, Met734, Ile 737, Glu893, Met894	
2Q7I (Askew et al., 2007)		Val716, Lys717, Val730, Gln733, Met734, Ile 737, Gln738, Glu893, Met894	Glu897, Lys720
2Q7K (Askew et al., 2007)		Val716, Lys 717, Gln733, Met734, Ile 737, Gln738, Glu893, Met894	Glu897, Lys720
2QPY (Estebanez-Perpina et al., 2007)		Val713, Val716, Lys720, Val730, Gln733, Met734, Gln738, Met894	Glu897, Lys720
4OEY (Hsu et al., 2014)		Val713, Val716, Val730, Gln733, Met734, Ile737, Gln738, Glu893, Met894	Glu897, Lys720
4OEZ (Hsu et al., 2014)		Val716, Phe725, Met734, Ile 737, Gln738, Glu893, Met894	Glu897, Lys720
4OFR (Hsu et al., 2014)		Val716, Phe725, Met734, Ile737, Gln738, Glu893, Met894	Glu897, Lys720, Asp731, Gln733
4OFU (Hsu et al., 2014)		Val713, Val716, Phe725, Met734, Ile737, Gln738, Glu893, Met894	Glu897, Lys720
4OH5 (Hsu et al., 2014)		Val713, Val716, Val730, Gln733, Met734, Ile 737, Gln738, Met894	Glu897, Lys720
4OHA (Hsu et al., 2014)		Val716, Val730, Gln733, Met734, Ile737, Gln738, Glu893, Met894	Glu897, Lys720
4OIL (Hsu et al., 2014)		Val716, Lys 720, Phe725, Met734, Ile737, Gln738, Glu893, Met894	Glu897, Gln733
4OIU (Hsu et al., 2014)		Lys720, Phe725, Met734, Gln738, Glu893	Glu897, Asp731, Gln733
4OJ9 (Hsu et al., 2014)		Val713, Lys720, Phe725, Met734, Ile737, Gln738, Met894	Glu897, Gln733
4OK1 (Hsu et al., 2014)	Trp741Leu, Arg760Ala	Val716, Gln733, Met734, Ile737, Gln738, Met894	Glu897, Lys720
4OKW (Hsu et al., 2014)	Trp741Leu, Arg760Ala	Val716, Phe725, Met734, Ile737, Gln738, Glu893, Met894	Glu897, Lys720, Gln733
4OKX (Hsu et al., 2014)		Val713, Val716, Phe725, Val730, Met734, Ile737, Gln738	Glu897, Lys720, Gln733
4OLM (Hsu et al., 2014)		Val713, Val716, Phe725, Val730, Met734, Ile737, Gln738	Glu897, Gln733

**FIGURE 5:**
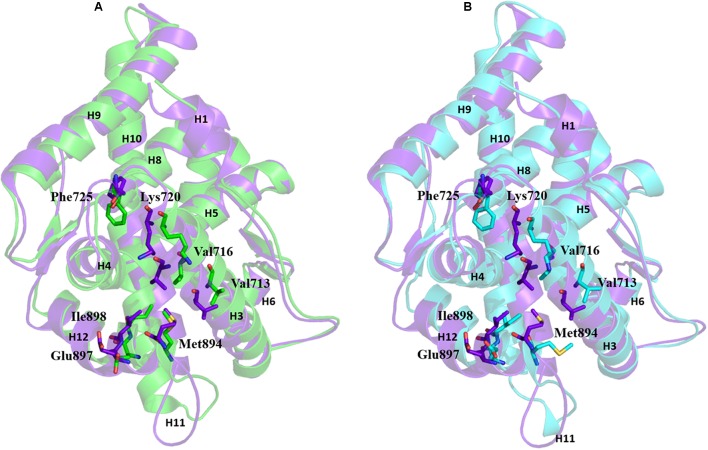
Overlay of WT-AR-R1881 in green and WT-AR-bicalutamide in purple **(A)**. Overlay of WT-AR-bicalutamide in purple and Mut-AR-bicalutamide in cyan **(B)**. The residues with different conformations in the AF2 site are presented as stick models.

Comparison of the AF2 site of the three representative structures (WT-AR-R1881, WT-AR-bicalutamide, and Mut-AR-bicalutamide) from the MD simulations shed light on critical residue displacements which prevent co-activator binding. Val713, Val716, Lys717, Lys720, Phe725, Met734, Met894, Glu897, and Ile898 were considerably different between WT-AR-bicalutamide and WT-AR-R1881 (**Figure 5A**). Among these residues, few had a considerable deviation in their side chain. The side chain distances of Glu897 (CD), Gln738 (CD), Met734 (SD), Val716 (O), Lys720 (CG) were 3.8, 4.2, 2.2, 2.0, and 2.2 Å, respectively, between the WT-AR-R1881 and WT-AR-bicalutamide. These residues also had different conformations between WT-AR-bicalutamide and Mut-AR-bicalutamide as depicted in **Figure 5B**, with respective side chain distances of Glu897 (CD), Gln738 (CD), Met734 (SD), Val716 (O), Lys720 (CG) as 3.2, 0.5, 1.8, 1.1, and 3.0 Å. Val716, Lys720, and Gln733 were previously experimentally proven to form a charge clump in the AF2 site, which interacts with co-activator proteins (Askew et al., 2007; Estebanez-Perpina et al., 2007; Estébanez-Perpiñá and Fletterick, 2009; Hsu et al., 2014). These residues had a remarkable deviation when comparing between the WT-AR-R1881 and WT-AR-bicalutamide structures in our data. Axerio-Cilies et al. (2011) experimentally proved that Met734 was pushed away from the AF2 site when bicalutamide binds AR. In addition, Zhou X.E. et al. (2010) demonstrated that Glu897 meaningfully interacted with a co-activator protein. Taken together, these previous results support our discovery: when bicalutamide binds WT-AR, Met734, and Glu897 move, which causes structural changes in H12. H12’s structural change renders the AF2 site not suitable for co-activator protein binding. Lys720, Glu897, Val716, and Met984 were found to play a major role in the binding of co-activator peptides (He et al., 2004; Hur et al., 2004).

### Electrostatic Potential Surface Analysis Revealed That Bicalutamide Binding Disturbed the Positive and Negative Charge Clump in the WT-AR AF2 Site

**FIGURE 6:**
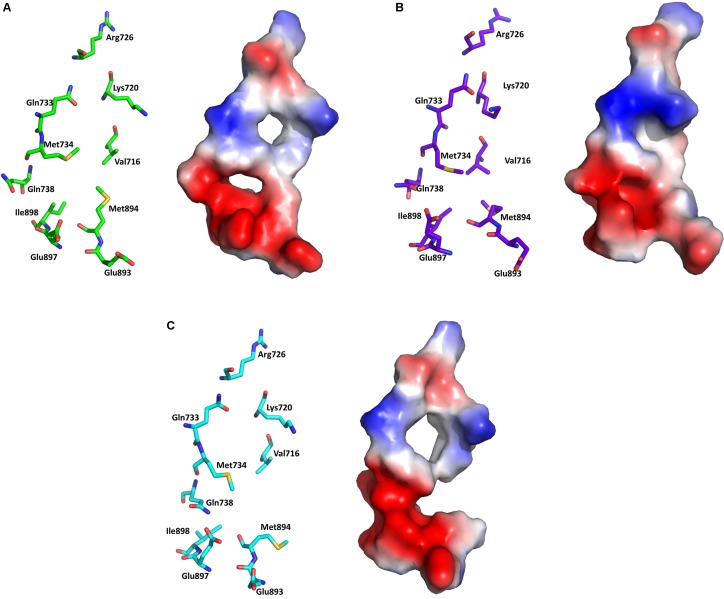
Electrostatic potential surface analysis for the AF2 site in WT-AR-R1881 **(A)**, WT-AR-bicalutamide **(B)**, and Mut-AR-bicalutamide **(C)**. The electrostatic potential surfaces are drawn in the right panels, where red indicates negative and blue indicates positive charges. The corresponding left panels show important residues in stick models.

Electrostatic potential surface analysis is one of the most powerful tools to study intramolecular interactions in a protein and intermolecular interactions between a protein and a small molecule (Sakkiah et al., 2013a). The electrostatic potential surface was calculated only for the critical residues in the AF2 site using PyMol (Baker et al., 2001). PyMol automatically generated the electrostatic potential map and smoothed out the local charge density of the nearby atoms (within 10 Å) without taking solvent screening effects into account^6,^^7^. The electrostatic potential surface of the AF2 site in WT-AR-R1881, WT-AR-bicalutamide, and Mut-AR-bicalutamide is shown in **Figure 6**. WT-AR-R1881 and Mut-AR-bicalutamide had very similar electrostatic potential surfaces in their AF2 site (**Figures 6A,C**), indicating the mutant residues turned the antagonist into an agonist. However, WT-AR-bicalutamide had a very different electrostatic potential surface (**Figure 6B**) compared with the other two structures due to structural changes in the AF2 site caused by the antagonist binding. Five residues (Val716, Lys720, Gln733, Gln738, and Met734) played an important role in bicalutamide binding induced WT-AR AF2 site structural changes. The binding of R1881 in the active site of WT-AR formed a positive (blue) and negative (red) binding region in the AF2 site (**Figure 6A**). Proximal residue contact closed the positive (caused by Gln733, Lys720, and Val716) and negative (caused by Met734, and Gln738) binding sites of the AF2 site in WT-AR-bicalutamide (**Figure 6B**). The critical residues in the Mut-AR-bicalutamide AF2 site (**Figure 6C**) showed a similar type of change compared with Mut-AR-R1881. Previously, it was experimentally proven that the charge clump was formed by residues Lys720 and Glu897 (Estebanez-Perpina et al., 2005; Tan et al., 2015). Co-activators can form hydrogen bond interactions with Lys720 and Glu897, leading to high binding affinity with WT-AR. These hydrogen bonds were distorted due to antagonist binding. Bicalutamide binding in the active site of WT-AR moved Lys720 and Glu897, disturbing the charge clump in the AF2 site and allowing for co-activator binding. Hence, the movement of Lys720, Val716, and Gln733 made the AF2 site unsuitable for co-activator proteins to bind together with bicalutamide. These computational findings give insight into the residues involved in the ligand induced conformational changes of the AF2 site.

## Conclusion

No structural details of WT-AR when bound by antagonists have been reported to date. Hence, we applied IFD and 1 μs long MD simulations to elucidate the bicalutamide binding induced structural changes of WT-AR’s AF2 site. IFD identified a suitable pose of bicalutamide in the ligand binding pocket of WT-AR. The best WT-AR-bicalutamide structure was selected based both on IFD score and on bicalutamide interactions with the critical residues in the ligand binding pocket of WT-AR. The complexes (WT-AR-R1881, WT-AR-bicalutamide, and Mut-AR-bicalutamide) were optimized by MD simulations using Amber 14. Our results clearly pinpointed residues Val716, Lys720, Gln733 and Met734, Gln738, and Glu897 as playing a pivotal role in the formation of the AF2 site in AR. Structural changes or movement of these residues due to bicalutamide binding changed the structure of the AF2 site, making it unsuitable for co-activator protein binding. The electrostatic potential map clearly revealed that the movement of these residues due to bicalutamide binding disturbed the positive and negative charge clump in the AF2 site of WT-AR. The positive clump in the AF2 site was distorted due to the movement of residues Lys720, Val716, and Gln733. Experimental validation is needed to confirm the mechanism by which bicalutamide binding induced WT-AR AF2 structural changes impact recruitment of co-factors.

## Author Contributions

SS and HH conceived the experiment(s). SS, BP, and WGe conducted the experiments. SS, BP, and WGo analyzed the results. SS, WT, HH, and RK wrote the manuscript. All authors reviewed and approved the manuscript.

## Disclaimer

The content is solely the responsibility of the authors and does not necessarily represent the official views of the Food and Drug Administration. The findings and conclusions in this article have not been formally disseminated by the US Food and Drug Administration (FDA) and should not be construed to represent the FDA determination or policy.

## Conflict of Interest Statement

The authors declare that the research was conducted in the absence of any commercial or financial relationships that could be construed as a potential conflict of interest.

## Footnotes

1 www.rcsb.org

2 www.schrodinger.com/induced-fit

3 https://www.schrodinger.com/protein-preparation-wizard

4 http://ambermd.org/doc12/Amber14.pdf

5 http://sts.bioe.uic.edu/castp/calculation.html

6 http://www.bccs.uni.no

7 http://www.bioinfo.no
