# Supplementary material for: Structural Changes Due to Antagonist Binding in Ligand Binding Pocket of Androgen Receptor Elucidated Through Molecular Dynamics Simulations
Source: Front Pharmacol. 2018 May 15;9:492. doi: 10.3389/fphar.2018.00492 (PMC5962723; doi:10.3389/fphar.2018.00492)
Supplement: Supplementary file 1 [file Table_1.DOCX]

Supplementary Material

Structural Changes Due to Antagonist Binding in Ligand Binding Pocket of Androgen Receptor Elucidated through Molecular Dynamics Simulations

Sugunadevi Sakkiah, Rebecca Kusko, Bohu Pan, Wenjing Guo, Weigong Ge, Weida Tong, and Huixiao Hong*

*** Correspondence:** Huixiao Hong: [Huixiao.hong@fda.hhs.gov](mailto:Huixiao.hong@fda.hhs.gov)


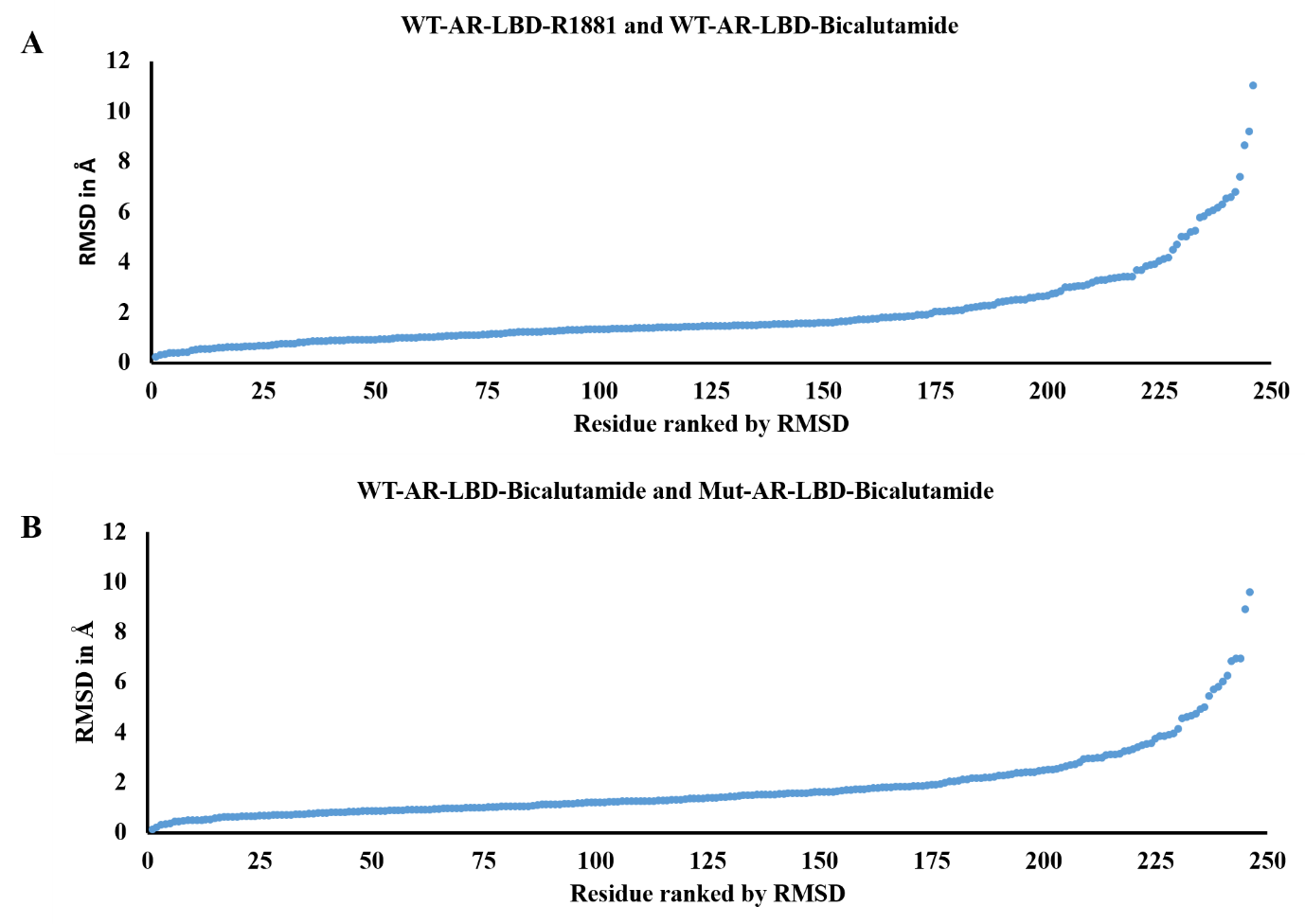


**Figure S1.** RMSD values of individual residues A) between WT-AR-R1881 and WT-AR-Bicalutamide, and B) between WT-AR-Bicalutamide and Mut-AR-Bicalutamide.

**Table S1:** RMSD values between WT-AR-R1881 and WT-AR-Bicalutamide

| **Site** | **Residue** | **RMSD** (Å) | **2ᵒ Structure** |
| --- | --- | --- | --- |
|  | Ser888 | 11.0289 | L (H10-H12) |
|  | Val887 | 9.2123 | L (H10-H12) |
|  | Val889 | 8.665 | L (H10-H12) |
|  | Asn848 | 7.4066 | L (H9-H10) |
|  | Asp890 | 6.7877 | L (H10-H12) |
|  | Pro849 | 6.5788 | L (H9-H10) |
|  | His885 | 6.5398 | L (H10-H12) |
|  | Met886 | 6.2951 | L (H10-H12) |
|  | Lys847 | 6.1831 | L (H9-H10) |
|  | Lys845 | 5.9871 | H9 |
|  | Arg846 | 5.8349 | L (H9-H10) |
|  | Ser884 | 5.7833 | L (H10-H12) |
|  | Thr850 | 5.2661 | H10 |
| LPB | Phe891 | 5.1968 | L (H10-H12) |
| AF2 | Glu893 | 5.0181 | L (H10-H12) |
|  | Pro892 | 4.6991 | L (H10-H12) |
|  | Cys844 | 4.4944 | H9 |
| LPB | Thr877 | 4.175 | H10 |
|  | Ala896 | 4.1308 | H12 |
|  | Lys883 | 4.0526 | L (H10-H12) |
|  | Ile882 | 3.9334 | L (H10-H12) |
|  | Phe878 | 3.8947 | H10 |
|  | Leu881 | 3.8524 | H10 |
|  | Ser851 | 3.6946 | H10 |
|  | Val901 | 3.6836 | H12 |
|  | Gln902 | 3.436 | H12 |
|  | Ser900 | 3.4236 | H12 |
|  | Val903 | 3.4183 | H12 |
| AF2 | Met894 | 3.4027 | L (H10-H12) |
|  | His714 | 3.3814 | H3 |
|  | His874 | 3.3462 | H10 |
| AF2 | Lys717 | 3.3078 | H3 |
|  | Ser908 | 3.2857 | H12 |
|  | Gly909 | 3.2567 | L (H12-S) |
| LPB | Leu873 | 3.2014 | H10 |
|  | Pro904 | 3.1104 | H12 |
|  | Asp819 | 3.0671 | L (S-H9) |
|  | Ser782 | 3.0474 | H7 |
| AF2 | Val713 | 3.0367 | H3 |
|  | Ser853 | 3.0143 | L10 |
| LPB | Phe876 | 2.9945 | H10 |
| LPB | Met895 | 2.8542 | H12 |

*LBP – Ligand binding pocket; AF2 – Activation Function Site 2

**Table S2: RMSD values between WT-AR-Bicalutamide and Mutant-AR-Bicalutamide**

| **Site** | **Residue** |  | **RMSD** (Å) | **2ᵒ Structure** |
| --- | --- | --- | --- | --- |
|  | Val887 |  | 9.5906 | L (H11-H12) |
|  | Ser888 |  | 8.9086 | L (H11-H12) |
|  | Val889 |  | 6.946 | L (H11-H12) |
|  | Asp890 |  | 6.9381 | L (H11-H12) |
|  | Met886 |  | 6.837 | H 11 |
|  | His885 |  | 6.279 | H 11 |
|  | Ser884 |  | 6.0391 | H 11 |
|  | Ile882 |  | 5.8166 | L (H10end-H11) |
|  | Leu881 |  | 5.7139 | H 10 |
|  | Lys883 |  | 5.4468 | L(H110-H11) |
|  | Phe878 |  | 5.0187 | H 10 |
| LBD | Phe 891 |  | 4.9405 | L (H11-H12) |
| LBD | Thr 877 |  | 4.7544 | H 10 |
|  | Pro892 |  | 4.6597 | L (H11-H12) |
| AF2 | Glu893 |  | 4.6198 | H 12 |
|  | His874 |  | 4.5505 | H 10 |
|  | Ser851 |  | 4.1535 | L (H9-H10start) |
| AF2 | Lys717 |  | 3.8984 | H 3 |
|  | Ala896 |  | 3.8666 | H 12 |
| LBD | Leu 873 |  | 3.8492 | H 10 |
| LBD | Leu880 |  | 3.7553 | H 10 |
|  | His714 |  | 3.5772 | H 3 |
|  | Asp879 |  | 3.5422 | H 10 |
| AF2 | Val713 |  | 3.4868 | H 3 |
|  | Leu907 |  | 3.3378 | H 12 |
|  | Ser778 |  | 3.2782 | H 6 |
|  | Ser853 |  | 3.2608 | H 10 |
|  | Ser908 |  | 3.1425 | H 12 |
|  | Ala870 |  | 3.1263 | H 10 |
| AF2 | Val716 |  | 3.1232 | H 3 |
|  | Gln875 |  | 3.0825 | H 10 |
|  | Lys912 |  | 2.9998 | S |
|  | Ser900 |  | 2.9909 | H 12 |
|  | Ser782 |  | 2.9711 | H 7 |
| LBP | Phe876 |  | 2.9501 | H 10 |
| AF2 | Met894 |  | 2.9445 | H 12 |
|  | Pro682 |  | 2.8081 | L (H1-H3) |

*LBP – Ligand binding pocket; AF2 – Activation Function Site 2; LBD – Ligand Binding Domain
